# Strategies to Better Target Fungal Squalene Monooxygenase

**DOI:** 10.3390/jof7010049

**Published:** 2021-01-13

**Authors:** Alia A. Sagatova

**Affiliations:** Department of Biochemistry, Albert Einstein College of Medicine, Bronx, NY 10461, USA; alia.sagatova@einsteinmed.org; Tel.: +1-718-430-4119

**Keywords:** squalene monooxygenase, squalene, 2,3-oxidosqualene, fungal infections, resistance mutations, dermatophytes, X-ray crystal structures, terbinafine, NB-598, antifungals, ergosterol, ergosterol biosynthesis pathway, antimycotics

## Abstract

Fungal pathogens present a challenge in medicine and agriculture. They also harm ecosystems and threaten biodiversity. The allylamine class of antimycotics targets the enzyme squalene monooxygenase. This enzyme occupies a key position in the sterol biosynthesis pathway in eukaryotes, catalyzing the rate-limiting reaction by introducing an oxygen atom to the squalene substrate converting it to 2,3-oxidosqualene. Currently, terbinafine—the most widely used allylamine—is mostly used for treating superficial fungal infections. The ability to better target this enzyme will have significant implications for human health in the treatment of fungal infections. The human orthologue can also be targeted for cholesterol-lowering therapeutics and in cancer therapies. This review will focus on the structural basis for improving the current therapeutics for fungal squalene monooxygenase.

## 1. Fungi—A Growing Problem

The fungal kingdom is incredibly large and diverse [1] with fungi occupying important roles in our environment, ecosystems, and our daily lives [1]. Amongst diversity, the odds are that some fungal organisms would be pathogenic to animals and plants. Indeed, several hundred fungal species are pathogens. In healthy people, pathogenic fungi can cause superficial infections. However, those with weakened immune systems are at a far greater risk of both superficial and invasive infections. In 2017, it was estimated that fungal infections cost $7.2 billion to the healthcare system in the United States, with $4.5 billion accounting for hospitalizations with the rest covering outpatient visits [2]. The same study showed that candidiasis and aspergillosis accounted for a large proportion of the hospitalization costs, whereas the intended purpose of over half of the outpatient visits was for treatment for dermatophyte infections of the skin, hair, and nails with an estimated total cost of $802 million [2].

In agriculture, major crops such as wheat, rice, maize, potatoes, and soybeans are susceptible to fungal phytopathogens. Millions of tons of crops are destroyed by fungal phytopathogens every year. Fisher et al. estimate that the crops lost to fungi could feed ~8.5% of the world’s population for a year [3]. Crop loss due to fungi is exacerbated by the use of monocultures; one prominent example of this is the Cavendish banana, which is susceptible to the *Fusarium* fungi. These fungi have been demolishing banana plantations in Asia for a long time and just last year, it was reported to spread to Latin America, threatening the global banana supply and driving up banana prices. Such threats to the global food security have resulted in increasing usage of antifungal pesticides [4]. Heavy use of pesticides comes at a cost—some fungi can develop resistance and, in the case of *Aspergillus fumigatus*, cross-resistance to medical antifungal drugs has been reported [5]. In addition, there are concerns over the impact of azole pesticides on the endocrine system [6].

There is also an emergence of infectious diseases caused by fungi in natural habitats that devastate plants and animals, damage ecosystems, and threaten biodiversity [3]. In 2006, a new disease caused mass mortalities in a population of bats in North America. The fungal pathogen *Geomyces destructans* was identified to be responsible for this disease, termed the “white-nose syndrome” as bats had fungi growing on their snouts and wings during hibernation [7]. This disease caused a 70% decline in the affected bat populations [8]. Ecosystems in parts of Central America were reshaped by the loss of about 40% of the amphibian population due to a skin-infecting fungus *Batrachochytrium dendrobatidis* [9]. This pathogen was originally identified in the 1970s in southern Mexico. It is now found on all continents with amphibian populations [10].

Climate change is one of the drivers impacting the dynamics and distribution of fungal organisms. One recent example of this is the newly emergent drug-resistant pathogen *Candida auris*, an organism that was not recorded to have infected humans until 2009 [11]. Most fungi grow better at low temperatures, for example, *B. dendrobatidis* does not grow well above 25 °C. Thus, it easily infects cold-blooded amphibians. Casadevall et al. argue that *C. auris* was driven by climate change to grow better at higher temperatures and, as a result of crossing this “temperature barrier,” became pathogenic for humans [12]. Given the size of the fungal kingdom and the growing impact of climate change, the probability of such an event occurring again is high.

Fungi pose a threat to human existence, which is likely to get worse in the coming years. Thus, treatment of fungal infections will play an even more crucial role in medicine, the agricultural industry, and biodiversity preservation as time goes on. In the following sections, the antifungal drugs will be discussed with particular focus on the allylamine antifungals and the enzyme they target, squalene monooxygenase (Erg1, SM—an abbreviation for the mammalian homolog). In 2019, the structure of the human homolog of this enzyme was determined by X-ray crystallography. This provided homology modeling opportunities that would allow for the design of better inhibitors for fungal enzymes [13].

## 2. Blocking Sterol Biosynthesis

**Figure 1:**
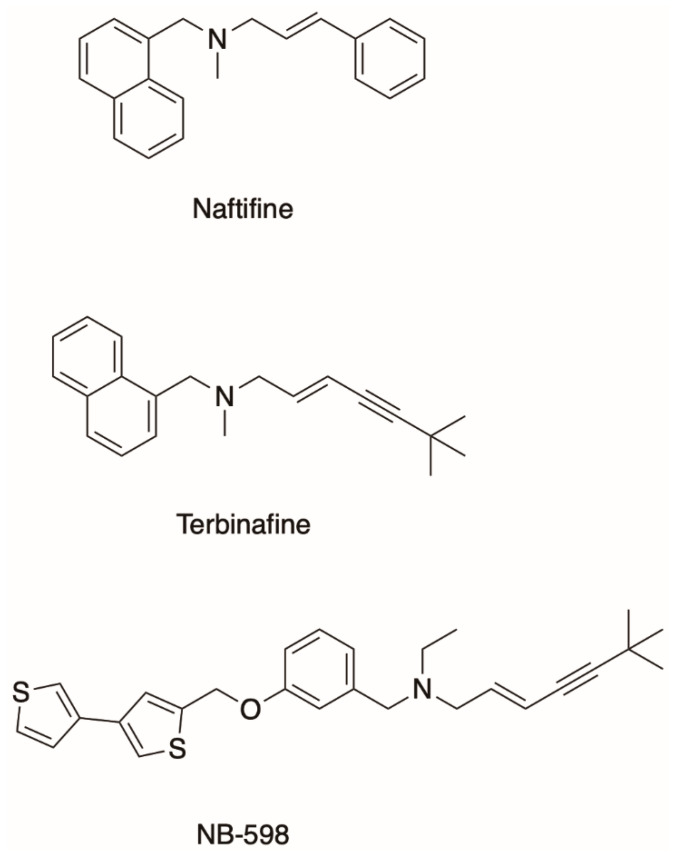
Allylamines terbinafine and naftifine and the human squalene monooxygenase inhibitor NB-598.

To combat fungal infections, the two classes of antifungals that target the mevalonate pathway to block fungal sterol/ergosterol production are the azole drugs (e.g., fluconazole, posaconazole) and the allylamines (e.g., terbinafine and naftifine; Figure 1). Ergosterol depletion changes the composition of the lipid bilayer altering the fluidity of the cell membrane and inhibits fungal growth [14]. This inhibition also allows for the accumulation of toxic ergosterol precursors in the fungal cell. The azoles target the fungal enzyme lanosterol 14 α-demethylase and have been very successful in the clinical and agricultural settings. However, there are still challenges to overcome such as toxicity, antifungal drug resistance, and the emergence of cross-resistance between agricultural and medical azoles [15]. Still, advances are being made, and the latest generation of azoles, the tetrazoles, are currently in clinical trials. VT-1161 is currently in phase III clinical trials for the treatment of recurrent vulvovaginal candidiasis [16,17]. VT-1598 is in phase I trials for the treatment of *C. auris* infections [18]. This progress with the development of the azole drugs has partially been driven by the need for novel antifungals in the medicine and agriculture and partially by extensive functional and structural characterization of lanosterol 14 α-demethylase from *Saccharomyces cerevisiae* [19,20], the pathogenic yeast *Candida albicans* [21], and others covered in a comprehensive review by Monk et al. [15].

**Figure 2:**
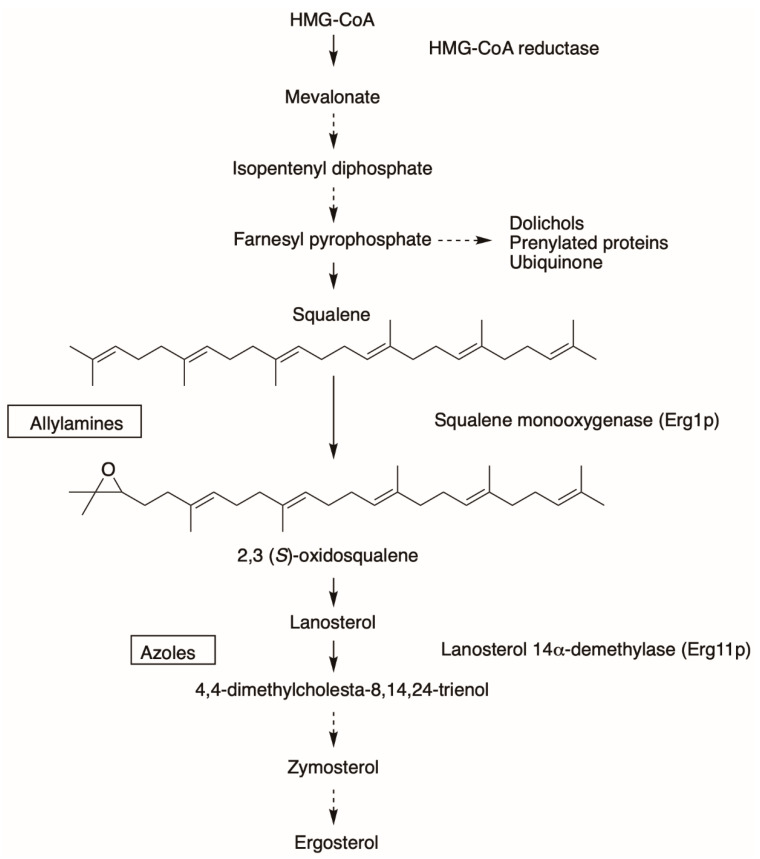
Ergosterol biosynthesis pathway. The simple arrow indicates one catalytic step from substrate to product and the dotted arrow represents presence of several additional catalytic steps.

On the other hand, the allylamine class of antifungals targets Erg1, a well-established but underdeveloped drug target [22]. SM is an endoplasmic reticulum-associated enzyme that catalyzes a rate-limiting step of cholesterol biosynthesis in mammals and ergosterol biosynthesis in fungi by converting squalene to 2,3-oxidosqualene (Figure 2). It is classified as a class E monooxygenase as it uses the flavin adenosine dinucleotide (FAD) as a cofactor, to receive electrons from NADPH-cytochrome P450 reductase (P450R) to carry out the epoxidation and the reduction of molecular oxygen to water [23]. There are only a handful of allylamine drugs, including terbinafine, naftifine, and butenafine. Terbinafine was derived from its predecessor naftifine by a substitution of a phenyl ring to a tert-butylacetylene group (Figure 1), improving the activity of the drug several-fold [24,25]. Allylamines have been successfully used in the treatment of superficial fungal infections such as tinea pedis (athlete’s foot), tinea capitis (scalp and hair), and onychomycosis (nail infection). These infections are very common. It is estimated that 20–25% of the global population have superficial mycoses [26]. These are generally caused by filamentous fungi that utilize keratin for growth, such as *Trichophyton* spp., *Microsporum* spp., and *Epidermophyton* spp.

## 3. Terbinafine

In general, dermatophytes are more susceptible to Erg1 inhibition than other fungi. This is partially due to the lipophilic nature of terbinafine which tends to localize at the sites of infection by dermatophytes such as skin, hair, and nails [25]. These fungi are also less tolerant to the accumulation of squalene [27]. In vitro studies show that terbinafine minimum inhibitory concentration (MIC) is higher for yeasts such as *C. albicans* and molds such as *Aspergillus fumigatus* [24,27]. Terbinafine can be effectively used in combination with other antifungals, but in the clinical setting, this must be done in a controlled manner as unmonitored use has the potential to select for multidrug-resistant fungal strains. A combination of the triazoles itraconazole or voriconazole with terbinafine has a synergistic effect against *A. fumigatus*, *Aspergillus flavus*, and *Aspergillus niger* [28]. Synergistic effects were observed in terbinafine/azole drug combinations for *Candida glabrata* strains with reduced azole susceptibility [29].

Although terbinafine is not used systemically as often as it is used topically, it is a well-tolerated drug. The absorption of the orally administered terbinafine hydrochloride is over 70% and it is mainly localized to the adipose tissue, skin, and nails [30,31]. The elimination of terbinafine from nails and skin takes a long time with it still detected in nails 30 weeks after cessation of treatment [32]. Approximately 70% of terbinafine is excreted by the kidneys into urine and the rest via the feces [31]. Terbinafine is subject to metabolism by the cytochrome P450 liver enzymes and one of the main metabolites found in blood plasma is the N-demethylated derivative of terbinafine [33]. Drug–drug interactions are important to consider in the oral administration of terbinafine as it has been found to inhibit CYP2D6 [34]. This liver enzyme metabolizes about 25% of drugs [35]. However, terbinafine was not found to inhibit CYP3A4, an important enzyme that is responsible for metabolizing ~50% of drugs [36]. Some azoles, on the other hand, can have stronger interactions with liver enzymes, for example, ketoconazole is a potent inhibitor of CYP3A4 [37].

Terbinafine has a much lower binding affinity for the human SM than for Erg1 [27,38]. On the contrary, NB-598 is a very potent inhibitor of the human SM but not of Erg1 (Figure 1) [13]. SM has been proposed as a target for cholesterol-lowering medications with the potential for fewer side effects than the current gold standard for treatment of hypercholesterolemia statins which inhibit HMG-CoA reductase. Fewer side effects would result from targeting the mevalonate pathway at a later step after the formation of isoprenoids required for the biosynthesis of dolichol and prenylated proteins. There is also a growing body of work that links SM to cancer [39]. One of the first cancer types to have links to SM was hepatocellular carcinoma, where SM was found to be overexpressed [40]. More recently, NB-598 was found to halt neuroendocrine cancer cell replication via inhibition of SM, due to a toxic effect of squalene accumulation [41]. In addition to the synthetic SM inhibitors such as NB-598 and compound-4, there are some natural ones like garlic extract compounds and resveratrol found in wine. There are no SM inhibitors currently in clinical use. For a comprehensive review of mammalian SM and its relevance to human health please refer to the review by Chua et al. [42].

The good pharmacokinetic profile of terbinafine makes it attractive for use outside of medicine. For example, the efficacy of terbinafine as a treatment for *Batrachochytrium dendrobatidis* infections in the amphibians was tested on alpine tree frogs. Unfortunately, terbinafine treatments did not cure the mycoses in these frogs [43]. Efforts into preserving the little brown bat population have been made by determining safe dosages of terbinafine [44]. Another study developed a subcutaneous implant with terbinafine and monitored its release in a saline solution. Although the terbinafine was released over 28 weeks at therapeutic concentrations, this was not repeated in an animal study [45].

## 4. Squalene Monooxygenase—Fine-Tuning Sterol Homeostasis

The sterol biosynthesis pathway is highly regulated. Several mechanisms are in place to prevent the accumulation of free sterols, which are toxic to the cells. Transcriptional regulation as well as regulation through the rate-limiting enzyme HMG-CoA reductase are key mechanisms for controlling the production of sterols [46,47]. SM plays a role in this process, fine-tuning sterol homeostasis in both fungi and mammals. Accumulation of the end product or one of the intermediates in the pathway results in SM degradation via the endoplasmic reticulum-associated protein degradation (ERAD) pathway.

In fungi, the Erg1 degradation was found to be regulated by lanosterol accumulation. *S. cerevisiae* cells treated with fluconazole, an inhibitor of lanosterol 14 α-demethylase, resulted in the accumulation of lanosterol and rapid degradation of Erg1 [48]. Really interesting new gene (RING) finger-type ubiquitin ligase Dao10 (E3 enzyme) targets Erg1 for proteasomal degradation [48]. Dao10 was reported to ubiquitinate the K311 residue of Erg1, this residue is located in the catalytic domain of the enzyme. It is unclear if there are other ubiquitination sites for Doa10 in Erg1. However, there is precedent to suggest that there may be ubiquitination sites other than lysines for Doa10p [49,50]. Doa10 requires the ubiquitin-conjugating enzymes (E2 enzymes) Ubc6p and Ubc7, with Ubc6 attaching the initial ubiquitin molecule to Doa10 substrates and Ubc7 carrying out polyubiquitination. Ubc6 was found to attach ubiquitin not only to lysine residues, but also to amino acids with hydroxyl groups (serine and threonine) thereby expanding ubiquitination sites for Doa10 [49,50].

The mammalian SM is targeted for degradation by ERAD, but unlike the fungal enzymes, it was shown to be sensitive to the accumulation of cholesterol and not lanosterol. SM has an N-terminal extension of 100 residues termed the N100 degron, which is absent in fungi. A 12-amino acid (Q62-L73) amphipathic helix was identified in this N100 degron domain, which is hypothesized to unfold and flip out of the lipid bilayer [51]. This exposes the loop for ubiquitination by the mammalian equivalent of Doa10, membrane-associated RING-CH-type finger 6 (MARCH6), an ER-localized E3 ubiquitin ligase [52]. Interestingly, four serine residues were found to be ubiquitinated by MARCH6, thus providing more evidence that Doa10 may ubiquitinate other residues in addition to the lysine residues in Erg1 [51].

## 5. Squalene Monooxygenase Expression and Purification

It has been over 20 years since *C. albicans* Erg1 was expressed in *S. cerevisiae* by Ryder et al. [53]. Similarly, human SM was also expressed in *Escherichia coli* and affinity-purified in 1999 [54]. The first experimentally determined crystal structure was released almost 20 years later [13]. This paucity of structural information is largely attributed to the fact that membrane proteins are notoriously difficult to purify and crystalize. This is reflected in the number of membrane protein structures deposited in the protein data bank (PDB) compared to the soluble proteins. Currently, the total number of entries in the PDB exceeds 170,000, and there are about 4300 entries for membrane proteins and of these only about 1200 are unique structures (mpstruc, https://blanco.biomol.uci.edu/mpstruc/). Often, the transmembrane domains of the membrane protein get truncated for ease of expression and purification. The choice of the detergent for solubilization of membrane proteins from the lipid bilayer in a monodisperse manner presents a challenge. Originally, SM was solubilized from rat liver microsomes using Triton-X100, which also acted instead of the supernatant protein factor needed to carry out squalene epoxidation [55]. It was subsequently discovered that supernatant protein factor contains a Sec14-like lipid-binding domain which can bind both squalene and 2,3-oxidosqualene. This was confirmed in X-ray crystal structures and this supernatant protein is now thought to ensure substrate flow to SM [56]. The fungal enzyme, on the contrary, is weakly stimulated by the soluble fraction and is inhibited by Triton-X100 [25,57].

Efforts have been made to hyperexpress full-length ScErg1, CaErg1, and human SM in the laboratory of Dr Brian Monk (Sagatova et al., unpublished). These constructs were successfully expressed in a well-established *S. cerevisiae* expression system designed for overexpression of membrane proteins in a hypersensitive host [58,59]. The successful hyperexpression was determined using Western blot in addition to the MIC susceptibility assays to terbinafine. However, membrane solubilization using a detergent that allows for the protein to be successfully purified and subsequently crystallized proved challenging. A non-ionic detergent n-octyl-β-d-glucoside solubilized ScErg1 as seen by size exclusion chromatography. Unfortunately, subsequent crystallization attempts were not successful.

For the current X-ray crystal structure of human SM, N-terminally truncated enzyme (118–574) was used for the crystallographic work, purified from *E. coli* and solubilized using the detergent 3-[(3-cholamidopropyl)dimethylammonio]-1-propanesulfonate (CHAPS) which had been determined to have the most stabilizing effect on SM using a thermal shift assay [13]. The authors confirmed the functionality of the truncated enzyme using a liquid chromatography–mass spectrometry method that they developed to directly detect the product 2,3-oxidosqualene. The conventional assay of squalene monooxygenase activity involves incubation of total cell extracts with a radioactively labeled substrate [11,25,57]. The LC–MS method is superior to the previously used method of thin-layer chromatography in terms of time, throughput, and accuracy. The LC–MS assay can be further utilized in developing the next-generation inhibitors of SM.

**Figure 3:**
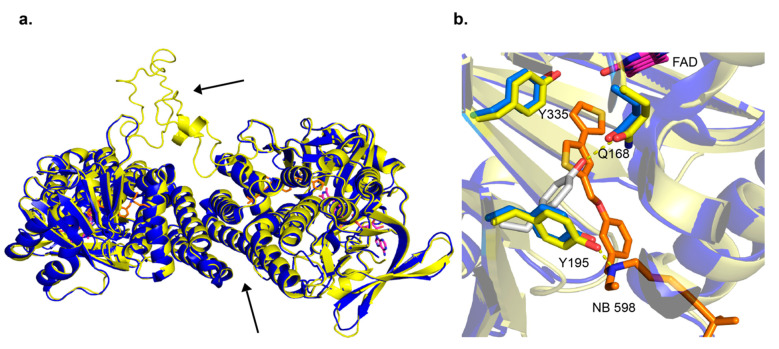
Overlay of human SM X-ray crystal structure and the *S. cerevisiae* Erg1 homology model. The homology model was downloaded from SWISS-MODEL with PDB ID 6C6N used as a template [60]. The ScErg1 is in yellow and SM (PDB ID 6C6P) is in blue. FAD and NB-598 are represented with sticks with carbons colored magenta and orange, respectively. (**a**) Two molecules were found in the asymmetric unit and the interface of C-terminal α-helices is indicated with an arrow. The extended loop of ScErg1 is shown with an arrow. (**b**) The residues Q168, Y195, and Y335 (Q63, Y90, and Y261, *S. cerevisiae* numbering) are shown as sticks. In gray is the Y195 residue position in the absence of the inhibitor (PDB ID 6C6R). The hydrogen bonds are shown as yellow dashed lines.

In addition to detergent screening, to obtain protein crystals of the fungal enzyme, one might consider the following strategies. The Erg1s of the yeasts *S. cerevisiae* and *C. albicans* have a longer loop between β-strands 6 and 7 (residues 109–139, *S. cerevisiae* numbering). This motif is much shorter in the human enzyme (residues 210–220), the dermatophytes, and the mold *A. fumigatus*. This short loop in the human enzyme facilitates crystal packing, i.e., the loops interact between unit cells. This extended loop in ScErg1 and CaErg1 may have presented a hindrance in crystal packing, thus truncating this region in ScErg1 or CaErg1 may prove successful (Figure 3). Elucidating the Erg1 crystal structure of a dermatophyte, such as *Trichophyton rubrum*, could be possible without such a truncation, as it does not have the elongated loop and has a relatively short N-terminus, which could be reduced by 32 residues to resemble the human SM structure.

## 6. Squalene Monooxygenase Structural Information

**Figure 4:**
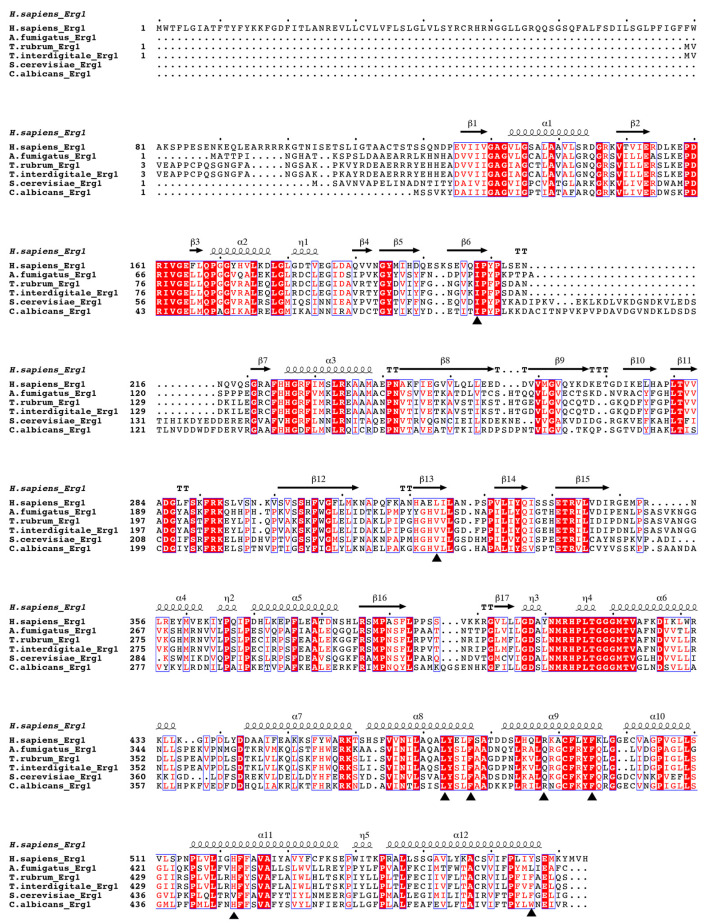
Sequence alignment of the human SM and fungal Erg1s. Sequence alignment was done using Clustal Omega [61] and the figure was generated using the ESPript 3.0 [62] online server using PDB ID 6C6P for secondary structure designation. The sequences were obtained in Uniprot *H. sapiens* (accession number Q145334), *S. cerevisiae* strain S288c (accession number P32476), *A. fumigatus* Z5 (accession number A0A0J5PRX5), *T. interdigitale* strain MR 816 (accession number A0A059JE48), *T. rubrum* (accession number Q4JEY0), *C. albicans* strain SC5314 (accession number Q92206). Conserved residues are highlighted in red and similar residues are shown in the red font. Mutations which confer reduced terbinafine susceptibility in dermatophytes *T. rubrum* and *T. interdigitale* are designated with triangles. The secondary structure is annotated as α for α-helices (curved line), β for β-strands (arrow), and η for 3_10_-helices, the dots on the top of the sequences appear every ten residues.

There had been no published structures for SM until last year when the human SM structure was published by Padyana et al. [13]. Three X-ray crystal structures were deposited into the PDB, one in complex with NB-598 (PDB ID 6C6P), one in complex with compound-4 (PDB ID 6C6N), and an unliganded structure (PDB ID 6C6R). The structures show a homodimer in the asymmetric unit, with C-terminal α-helices 11 and 12, residues 516–537 and 545–567, respectively, forming the interacting interface. Those C-terminal α-helices are putative membrane-associated domains, so it makes sense that they will form stabilizing hydrophobic interactions (Figure 3a). As shown by Padyana et al. in thermal stability assays, the SM (118–574) was more stable than the SM (118–488) by 4 °C. The FAD-binding domain was identified to have the GR2 Rossmann fold. The inhibitor-binding site was identified with both NB-598 and compound-4 binding in an extended conformation into a relatively hydrophobic binding pocket consistent with the isoprenoid substrate squalene. The clinically relevant ligand terbinafine was also modeled into the unliganded structure of SM. The authors identified interactions of the binding site and terbinafine and mapped some of the resistance mutations which occur in Erg1 [13] (discussed in later sections). This provides useful information as there is no X-ray crystal structure available for the fungal enzymes to date. The human SM structure is good for homology modeling and other computational techniques, as the substrate-binding site is relatively conserved among human SM, and the Erg1s of the dermatophyte fungi *T. rubrum* and *Trichophyton interdigitale*, and yeasts like *C. albicans* and *S. cerevisiae* (Figure 4).

A detailed in silico homology model was determined in 2011 (*S. cerevisiae* Erg1) using a crystal structure of p-hydroxybenzoate hydroxylase from *Pseudomonas fluorescens* (PDB ID 1PBE) and an aromatic hydrolase from *E. coli* (PDB ID 2QA1) [63]. Both these enzymes have FAD-binding domains but low sequence homology to the fungal enzyme. The ScErg1 model identified the FAD and the substrate-binding domains, including the characteristic GR2 Rossmann fold [64]. The authors used a 3D-Jury approach to get the overall 3D fold and Modeler was used for the missing loops and sidechains. Molecular dynamics simulations were done to model terbinafine binding to Erg1. In addition, they used a mutagenesis study to verify their findings [65]. Mutagenesis studies of the *S. cerevisiae* enzyme showed that G27S, G30S mutations reduced the activity of the enzyme in vitro. The authors hypothesized that this was due to the proximity of these residues to the FAD cofactor. These residues are conserved in the human and the dermatophyte *T. rubrum* enzymes (Figure 4). The SM structure by Padyana et al. shows that the SM-equivalent glycine residues are in 3–4 Å proximity to the FAD molecule [13]. Several residues important for terbinafine binding were identified in the in silico model. Among them was Y90, which showed that its hydroxyl group projected to make a 2.4 Å hydrogen bond with the amine group of terbinafine. In the structural model of SM, Padyana et al. found that the equivalent residue Y195 makes a hydrogen bond with the tertiary amine of NB-598 and compound-4, the only hydrogen bond between the enzyme and those inhibitors (Figure 3b) [13]. In silico model identified this important interaction of ScErg1 with terbinafine in the lipophilic binding cavity, which illustrates the power of such predictive models. Furthermore, the progress of computational techniques has been immense since that time and protein structure predictions are getting better and better. As an example, DeepMind’s artificial intelligence system called AlphaFold outperformed its competitors in predicting protein structures and achieved results that are very close to the experimentally determined structures in the Critical Assessment of Structure Prediction in 2020 [66].

Another interesting interaction found in the binding site of ligand-free SM (PDB ID 6C6R) is the change of conformation of Y195 to form a hydrogen bond with the amine of Q168. Both residues are conserved in the fungal enzyme (Figure 3b). This is a very important interaction, which is redirected when an inhibitor is bound. It is worth noting that a tertiary amine is present in all SM and Erg1 inhibitors. The importance of this interaction and the Y195 residue is highlighted by an ~90% loss of activity by SM when this tyrosine is mutated to phenylalanine [13]. Another important tyrosine is located close to the flavin moiety of FAD (PDB ID 6C6P). Y335 forms a hydrogen bond network with the N5 of flavin via a water molecule. This is also conserved in fungi, and if the side chain of it is rotated about 30 degrees, it can make a hydrogen bond to Y195. It is therefore tempting to hypothesize this residue network is involved in proton-coupled electron transfer to the FAD. However, it is not yet know how the binding of squalene affects the conformation of the residues in the binding cavity as there is no crystal structure with the substrate.

## 7. Terbinafine Resistance Mutations

There are several ways that fungi can become tolerant and less susceptible to antifungals such as their stress response mechanisms, drug efflux via efflux pumps, gene duplication, and mutations in the drug target gene [67]. In dermatophytes, the main mechanisms of resistance to terbinafine are through point mutations in Erg1. Some of the first mutations found in clinical isolates harbored L393F and F397L mutations in *T. rubrum* Erg1 [68,69]. The potency of the F397L mutation was demonstrated when the *S. cerevisiae* strain was transformed with *C. albicans* Erg1 with and without the equivalent mutation increasing the MIC90 more than 16-fold [69]. It is worth pointing out that when resistant clinical isolate strains are compared with the susceptible ones, the changes in MIC may not only be attributable to a particular mutation as other resistance/tolerance mechanisms may be in play.

These same mutations L393F and F397L were subsequently found in clinical isolates of *T. interdigitale* [70]. The F397 position of the residues changes to a less bulky side chain like leucine, isoleucine, and valine, and the L393 is mutated to either a bulky phenylalanine or a polar serine [71]. Mutagenesis studies carried out on ScErg1 to find strains less susceptible to terbinafine have found an equivalent residue mutation to F397L (F402L; Figure 4) as well several additional ones [65]. The structurally equivalent residues of SM are found in the inhibitor-binding pocket [13]. Since there is only one polar interaction between the binding cavity and the inhibitor, most of the mutations change the size of the hydrophobic side chains, thereby altering hydrophobic interactions.

Terbinafine only partially inhibits human SM. Recently, Padyana et al. reported an IC50 of 7.7 µM and 65% inhibition at 100 µM [13]. Terbinafine modeled into SM places the naphthalene ring close to residues I197 and L324, which overlaps with the smaller benzene ring of NB-598. In contrast, in dermatophytes, those residues are valines. The authors hypothesized that the smaller residues accommodate the presence of the bulky naphthalene moiety of terbinafine better than the bigger leucine and isoleucine residues. Interestingly, the frequently mutated residues F397 and L393 are located adjacently to those valines in the binding pocket, highlighting the importance of hydrophobic interactions in drug binding. F415I/V, H440Y, and Q408L mutations have been identified. These are located closer to the trimethyl end of the terbinafine molecule [71,72] when modelled into SM. However, these mutations do not confer reduced susceptibility to terbinafine to the same extent as L393F and F397L. Double mutants H440Y F484Y and I121M V237I have been reported and were classified as “low-level” resistance mutations [73]. The *T. rubrum* strain with the double mutations resulted in an MIC of 0.125 μg/mL in comparison with *T. rubrum* with the “high-level” L393F mutation (MIC > 8 μg/mL) and the susceptible strain (0.016 μg/mL).

## 8. Concluding Remarks

Considerable progress has been made concerning understanding squalene monooxygenase, including the identification of important differences between the fungal and the mammalian enzymes, their structural characterization, and the links of the human enzyme to cancer.

Overcoming resistance mutations in dermatophytes and improving the efficacy of allylamines in yeasts such as *C. albicans* and *A. fumigatus* will have significant benefits for medicine and possibly in agriculture. Elucidating the structure of the fungal enzyme will shed more light on how to better target Erg1 and develop better antifungals. In the meantime, the human SM crystal structure provides a good avenue for finding better ways to purify and crystalize the fungal enzyme and for providing homology models to aid in developing novel antifungals. Hydrophobic interactions play an important role in drug binding to SM. However, there are polar residues in the active site that can be targeted such as Q168 (SM numbering), a residue conserved in fungi. Increasing the water solubility of allylamines will allow for better distribution throughout the body and not mainly in the skin and adipose tissue. This may increase the efficacy of drugs in treating disseminated infections. The same may also be true for targeting SM. NB-598 is a potent inhibitor but its toxicity has prevented clinical trials. In dogs, administering NB-598 has resulted in a reduction of cholesterol, triacylglycerol, and LDL-cholesterol [74]. Unfortunately, dermatitis-like toxicity effects were observed. This effect was thought to be due to the accumulation of squalene in the skin. In a recent study, the effects of NB-598 were investigated in cynomolgus monkeys and repeated in dogs [75]. In addition to dermatitis, gastrointestinal toxicity was observed in monkeys to the point where the animals had to be euthanized after 3 or 4 days of dosing [75]. It was confirmed that squalene accumulation in the skin is the result of the co-localization of NB-598. Much more work needs to be done to develop next-generation inhibitors including consideration of more appropriate methods of drug delivery.

## Data Availability

All the data used here was publicly available in the form of published articles, crystal structures added to the PDB, and homology model deposited into the SWISS Prot database.
